# Efficient digitalization method for dental restorations using micro-CT data

**DOI:** 10.1038/srep44577

**Published:** 2017-03-15

**Authors:** Changhwan Kim, Seung Hoon Baek, Taewon Lee, Jonggun Go, Sun Young Kim, Seungryong Cho

**Affiliations:** 1 KAIST, Department of Nuclear and Quantum Engineering, Daejeon, 34141, Republic of Korea; 2 Guidance Dental, Buena Park, California, 90621, USA; 3 Kyung Hee University, Department of Conservative Dentistry, School of Dentistry, Seoul, 02447, Republic of Korea

## Abstract

The objective of this study was to demonstrate the feasibility of using micro-CT scan of dental impressions for fabricating dental restorations and to compare the dimensional accuracy of dental models generated from various methods. The key idea of the proposed protocol is that dental impression of patients can be accurately digitized by micro-CT scan and that one can make digital cast model from micro-CT data directly. As air regions of the micro-CT scan data of dental impression are equivalent to the real teeth and surrounding structures, one can segment the air regions and fabricate digital cast model in the STL format out of them. The proposed method was validated by a phantom study using a typodont with prepared teeth. Actual measurement and deviation map analysis were performed after acquiring digital cast models for each restoration methods. Comparisons of the milled restorations were also performed by placing them on the prepared teeth of typodont. The results demonstrated that an efficient fabrication of precise dental restoration is achievable by use of the proposed method.

Dental restoration is the repairing process of a damaged tooth, restoring it back to its normal appearance and function. Restoring the tooth with crown or inlay requires an accurate and precise impression of the prepared tooth. A conventional impression using elastomeric impression materials and cast of a stone replica has been used to copy the detailed surface shape of the prepared tooth and its relationship with the surrounding teeth and the soft tissue. Such conventional impression method has been extensively investigated and considered as the gold standard which presents accurate and reliable information about patients’ intraoral conditions^1^,^2^,^3^,^4^,^5^. On the other hand, this traditional method has several disadvantages such as laborious procedures and storage burden^5^,^6^,^7^. In addition, the accuracy of stone cast model is heavily dependent on the skills and experiences of a dental technician.

As an advanced option, digital impression system using optical scanner is now available in clinical dentistry. Digital impression is produced either by scanning the stone cast fabricated through a conventional impression procedure or by directly scanning the prepared tooth using an intraoral scanner. Restorations may be directly fabricated through the computer-aided design and computer-aided manufacturing using the digital impression, enabling the restoration of the damaged tooth in a single visit. Another workflow for crown or inlay restorations is to take a digital impression, transfer the scan data electronically, and fabricate the physical master cast model and the restorations in dental laboratory, which eliminates the need for the conventional impression and manual fabrication of master cast stone model^8^,^9^. These digital impression systems have advantages over conventional impression: an increase in patient comfort, higher time- and cost-efficiency, reducing random geometric errors according to the volume change of materials and user skill, giving cleaner environment, and saving of materials^10^,^11^,^12^,^13^. Moreover, a number of studies on digital impression reported that the accuracy of the digital impression is similar to that of the conventional impression^13^,^14^,^15^,^16^,^17^,^18^,^19^,^20^,^21^,^22^. However, digital impression system by either scanning the stone cast extraorally or scanning the teeth intraorally also has its own limitations. As for the scanning the stone cast extraorally, it still requires a fabrication of stone model so that random geometric errors from the manual work cannot be avoided and savings of materials cannot be expected despite of the advantage of relatively easy scanning procedure^11^,^12^. Although the digital impression system by direct scanning of tooth intraorally has relative advantages of simple workflow and savings of materials compared to the extraoral scanning method, it has a steep-learning curve to master^11^,^23^. In addition, the accuracy can be easily degraded by acquisition conditions^21^,^24^,^25^,^26^. For example, severe errors can occur if the received light signal does not correctly recover the surface information due to the non-uniform reflectivity of the scanning surfaces particularly when wet^27^. We would like to also note that, due to the anatomy of the natural dentition, dental impressions may bear undercuts of which the optical scanner cannot capture the surface data accurately.

As both digital impressions by scanning extraorally and intraorally have their cons and pros, a precise and convenient digital impression method for dental restoration is still desirable, which can mitigate the shortcomings and maximize the merits of each method. In this work, we propose a novel and efficient method to obtain the digital impression for dental restorations by use of micro-CT scanning of a conventional dental impression. With an image processing on the micro-CT scan data, the digital cast model for a dental restoration can be directly produced. The aim of this study was to check a feasibility of the proposed method and to evaluate the accuracy. We performed a phantom study using a typodont with prepared teeth and demonstrated that efficient fabrication of precise dental restoration is achievable by use of the proposed method.

## Methods

### Workflow of the proposed method

**Figure 1:**
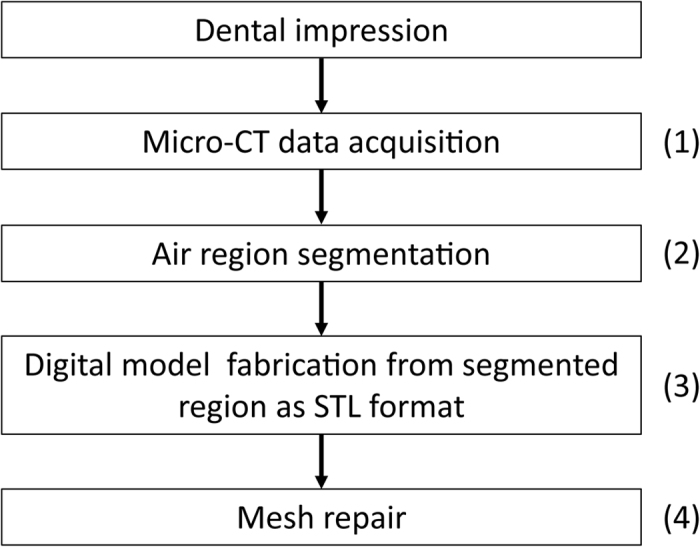
Pipeline of the proposed method.

In this section, we explain the proposed method of fabricating dental restoration based on micro-CT data of the dental impression. The key idea of the proposed method is that dental impression of patients can be accurately digitized by micro-CT scan and that one can make digital cast model from micro-CT data directly. As air regions of the micro-CT scan data of dental impression are equivalent to the real teeth and surrounding structure, one can segment the air regions and fabricate digital cast model in the STL format out of them. A conceptual workflow of the proposed method is summarized in Fig. 1.

After an impression acquisition from a patient (a typodont phantom in this study), the dental impression is scanned with a micro-CT system first. The reconstructed volume data of the dental impression is then saved in DICOM format. NFR Polaris-S90 (Nanofocusray, Jeonju, Korea) was used for micro-CT scanning in this work. The scanning parameters and system geometry are summarized in Table 1.

Air regions were then segmented from the DICOM data. As air regions represent teeth and surrounding structures, we extracted the regions by use of a threshold-based segmentation on the entire stack of DICOM data. The threshold value should be determined from the CT metrological point of view in such a way that accurate separation of the air regions from the impression materials can be achieved. In this work, we have empirically arrived at an optimum threshold value based on a ground truth dimension which is known a priori. More details will be explained in the phantom description part in the following.

Then, surfaces of the segmented air regions were extracted and converted into the digital model in the STL format. The digital model of the segmented regions was constructed by triangular meshes. For the converting procedures, we utilized 3D Slicer (http://www.slicer.org) which provides a free platform for biomedical research. More precisely, the module named Model Maker, based on marching cube algorithm^28^, in 3D Slicer was used to create 3D surface model from the segmented image data. In addition, a joint smoothing process was also applied to relieve the pixelated appearance caused by the inter-slice discontinuity in the DICOM images.

After creation of a digital dental cast model, we applied several mesh-repair processes to improve the quality of meshes and reduce the residual artifacts by use of Geomagic studio software (3D System, Morrisville, NC, USA). Spikes artifact due to the noise in CT data was mitigated, and unwanted structures such as air-bubbles in the dental impression were removed. By polishing polygon meshes, we obtained a reliable digital cast model for accurate dental restoration.

### CT scan techniques

**Figure 2:**
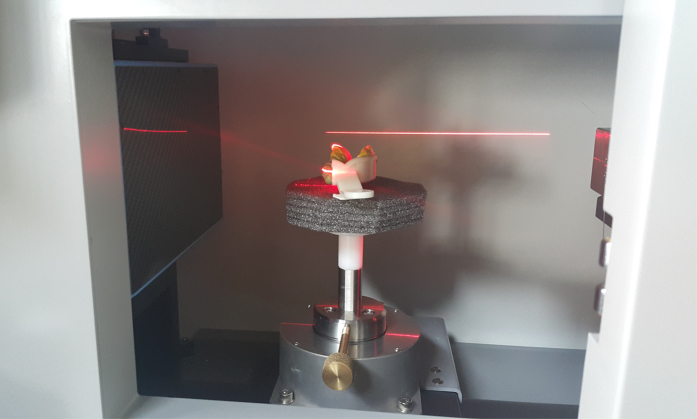
Scan method to minimize the cone-beam artifact.

The key point of the proposed method is that the intraoral information recorded as a form of physical dental impression can be efficiently digitalized by use of a micro-CT system. Therefore, image quality of the reconstructed CT images is critically important. In CT imaging, there are several image artifacts that can occur with their severity depending on the scanning conditions. As artifacts can degrade accuracy of the image and the related intraoral information, it is important to find the optimal scan conditions that can circumvent image artifacts if possible. Additionally, unavoidable image artifacts must be reduced by use of appropriate correction techniques. In this work, we placed the dental impression in such a way that cone-angle artifacts can be minimized. Cone-angle artifacts are caused by inconsistent data in the off-midplanes. In a circular CBCT scanning geometry, only the image on the midplane where the x-ray source trajectory lies can be exactly reconstructed. Off-midplanes do not satisfy the so-called Tuy’s condition which guarantees exact image recovery in CBCT^29^. This is related to the diverging nature of the x-ray cone-beam, and constitutes an unavoidable physical factor that leads to image artifacts and inaccuracy. The severity of cone-angle artifacts depends on the cone-angle and also on the axial variations of the anatomical components of the scanned object. The larger the cone-angle becomes and the more axial variation the imaged object anatomy becomes, the stronger image artifacts would show up after reconstruction. Usually, for relatively small cone-angles, *e.g.*, 5 degrees, and for objects of relatively uniform anatomy the cone-angle artifacts are negligible and do not degrade image accuracy to a practical sense. Dental impression is of relatively uniform material, *e.g.,* polyvinylsiloxane or polyether on top of a tray. Therefore, we placed the dental impression so that its arc plane is parallel to the scanning midplane and that the cone-angle associated with the axial FOV coverage can be minimized as shown in Fig. 2.

Beam-hardening artifacts are caused by polychromatic nature of the x-ray source. Since higher energy photons are less attenuated than lower energy photons, reconstructed attenuation coefficients of a scanned object would depend on the local arrangement of its anatomical components. Beam-hardening artifacts show up as streaks and shadows in the reconstructed images. Various beam-hardening artifact correction methods have been investigated, and we utilized a linearization method which is one of the pre-reconstruction method for beam-hardening correction^30^,^31^,^32^. By applying polynomial fitting to the measured attenuation as a function of thickness, one can generate a conversion scheme that corrects for the non-linearity in the reconstructed images due to beam-hardening artifacts. We have not only developed a beam-hardening correction approach but also selected the x-ray tube voltage so that intrinsic beam-hardening can be reduced. Beam-hardening artifacts dominate when the x-ray tube voltage is relatively low. However, image contrast is enhanced at low tube voltage because more photoelectric interaction would be involved than Compton interaction. Therefore, we have empirically determined the x-ray tube voltage to be 60 kVp as an optimum condition balancing between image contrast and beam-hardening artifacts.

### Phantom

**Figure 3:**
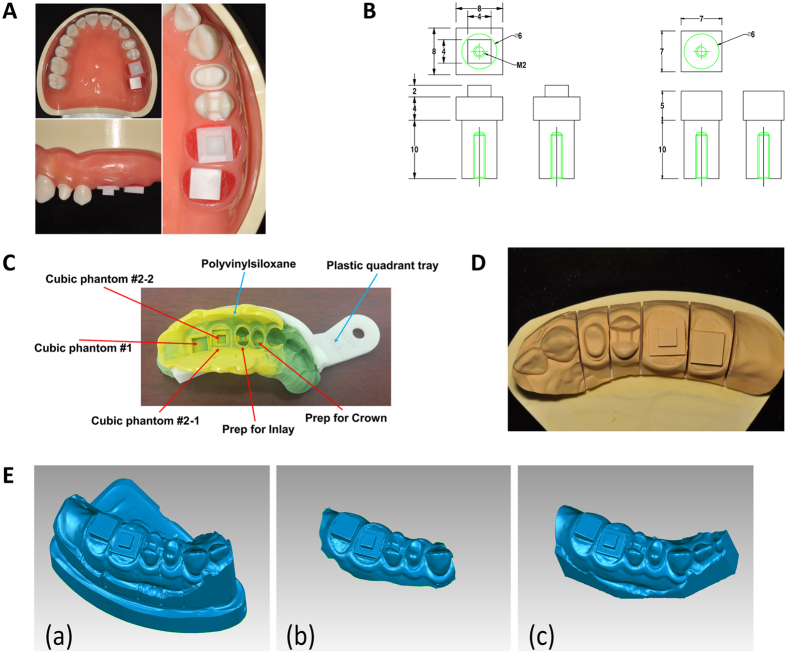
Phantom information for the comparison study. (**A**) Typodont model with prepared teeth used in this work. (**B**) Specifications of each rectangular-shape phantom. (**C**) Dental impression of the master phantom in this work. (**D**) Plaster cast model of the master phantom in this work. (**E**) The digital cast model for each method. (a) Extraoral optical method, (b) Intraoral optical method, and (c) proposed method.

A typodont model (D85DP-500B.1, Nissin Dental, Kyoto, Japan) with four prepared teeth was used for the master model in this work as shown in Fig. 3A. We fabricated mesio-occluso-distal inlay on #13 and full crown on #12 of the typodont model. We have additionally made two rectangular-shaped teeth phantoms for CT metrological purposes, and placed phantoms on #’s 14 and #15 respectively by them as shown in Fig. 3A. Detailed dimensional specifications of the two rectangular-shaped teeth phantoms are described in Fig. 3B.

### Comparison study

We conducted a study to check the feasibility of the proposed method and to compare its accuracy with the existing methods. We utilized the typodont master phantom (see section 2.3) for the comparison study and applied each method as following:

1. Extraoral optical method: After acquiring a dental impression with polyvinylsiloxane and plastic quadrant tray (KOD, Seoul, Korea) (Fig. 3C), we made the plaster cast model (Fig. 3D). The cast model was scanned using the desktop laboratory scanner (D800, 3Shape A/S, Copenhagen, Denmark).
2. Intraoral optical method: The master phantom was scanned using an intraoral scanner (Cerec Omnicam, Sirona, Bensheim, Germany).
3. Proposed method: After acquiring a dental impression, the impression was scanned using a micro CT. Based on the CT image, digital cast model for dental restoration has been constructed.

For each method, resulting digital cast model was represented in the STL format as shown in Fig. 3E. After acquiring three digital cast models in the STL format, we analyzed them in two different ways: Actual measurement of the rectangular phantoms’ side lengths and a deviation map analysis were performed. Furthermore, we fabricated the inlay and crown and qualitatively assessed the resulting restorations of each method by placing them on the typodont model.

## Results

We checked the dimensional accuracy of each digital cast model by measuring the side length of two rectangular phantoms. For a measurement, we utilized the Geomagic Studio software and its point-to-point distance measure function. Two vertices at the end of each corner were selected and we directly measured the Euclidian distance between them. Measurements were performed 10 times by single operator to avoid interpersonal measurement errors. Means, standard deviations, and 95% confidence intervals (CI) were calculated, and the results are summarized in Table 2. The extraoral optical method showed the dimensional accuracy of (6.987 ± 0.007 mm), (7.987 ± 0.008 mm), and (3.988 ± 0.009 mm) in Phantom #1, Phantom #2-1, and Phantom #2-2, respectively. The digital cast of intraoral optical method offered the dimensional accuracy of (6.981 ± 0.009 mm), (7.984 ± 0.008 mm), and (3.985 ± 0.007 mm). The digital cast model of the proposed method showed the dimensional accuracy of (6.984 ± 0.009 mm), (7.984 ± 0.009 mm), and (3.986 ± 0.007 mm). Even though differences of the dimension were found among methods, there were no significant discrepancies among them. The results indicated that our proposed method is comparable with the extraoral optical and intraoral optical methods in terms of dimension accuracy.

**Figure 4:**
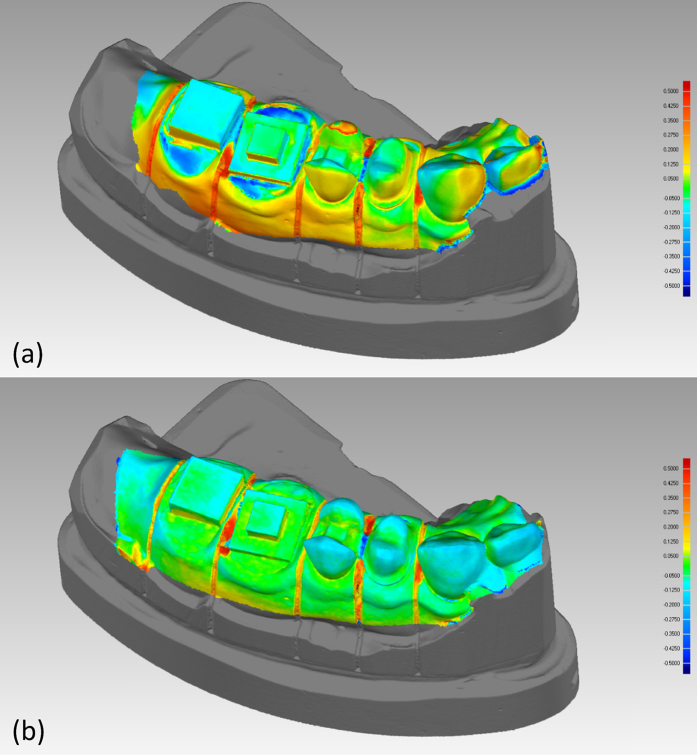
Deviation map of digital cast model. (**a**) Intraoral optical method and (**b**) proposed method. Deviations measured from reference (Extraoral optical method).

Deviation map analysis was also performed. Because the extraoral optical method is considered the most commonly used digitalization method, we acquired deviation maps with respect to the conventional digital cast model. Table 3 and Fig. 4 illustrate the three-dimensional deviation maps in pseudo-colors and the statistical results of intraoral optical method and the proposed method, respectively. For each case, the discrepancies range from −0.5 mm (blue) to 0.5 mm (red). Negative deviation (navy blue to light blue) represents under-estimation compared to the reference digital cast model. Positive deviation (red to yellow) represents over-estimation. 0.1 mm (−0.05 mm to 0.05 mm) was selected as an acceptable marginal range as shown in light green for crown restoration. Results of the deviation map analysis demonstrated that the proposed method offers relatively smaller deviations compared to the intraoral optical method not only on the teeth surface but also in the gingiva structures.

**Figure 5:**
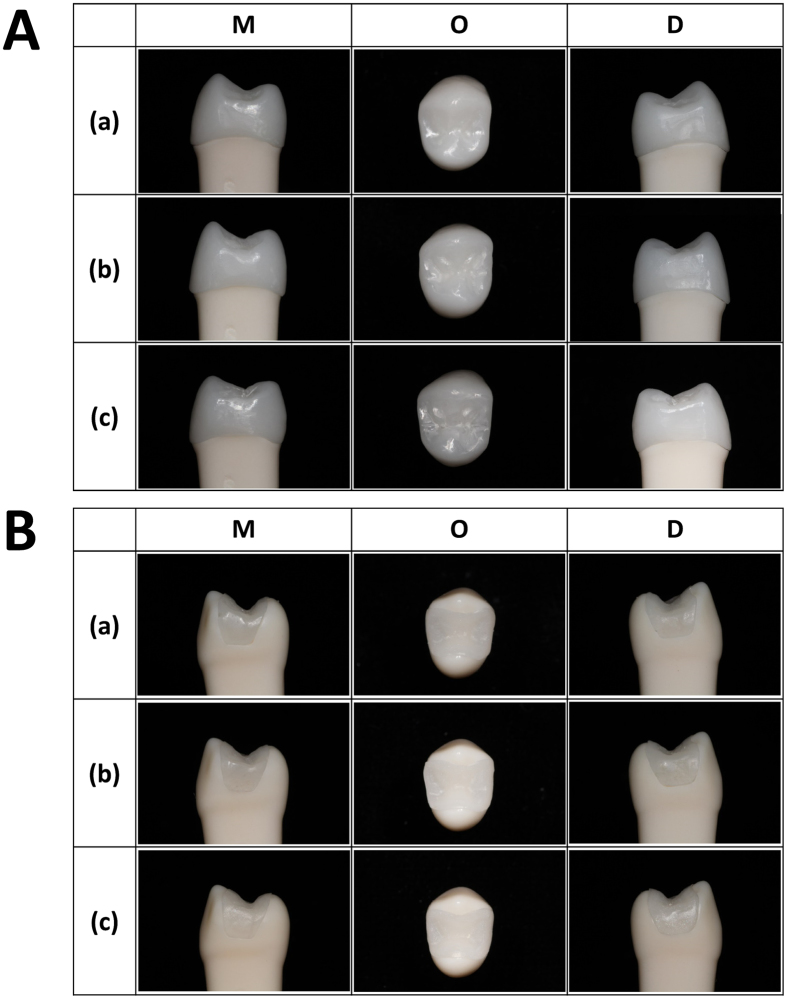
Mesial, Occlusal, and Distal view of final (**A**) crown and (**B**) inlay restorations on the typodont model. (a) Extraoral optical method, (b) Intraoral optical method, and (c) proposed method.

We also evaluated resulting restorations of each method. Digital cast models by each method were fed into the design software (Exocad, Darmstadt, Germany) and the milling machine (Protech Innotion, Cheonan, Korea), which fabricated restorations for mesio-occluso-distal inlay on #13 and full crown on #12. We utilized composite resin (B&D Dental Technologies, West Valley City, Utah, USA) for inlay and zirconia (B&D Dental Technologies, West Valley City, Utah, USA) for crown that are currently used for dental restorations. After constructing milled restorations, we placed them on the typodont phantom to qualitatively compare and check the marginal adaptation of each method. Figure 5 displays mesial, occlusal, and distal view of final crown and inlay restorations sitting on the typodont model by each method. The results shows that our proposed method is comparable with the existing methods in terms of appearance and marginal adaptation.

## Discussion

This study investigated the feasibility and accuracy of the micro-CT based dental restoration method for the first time to the best knowledge of the authors. The results of the comparison study showed that the proposed method is suitable for fabrication of dental restorations in terms of dimensional accuracy. Although the study was limited to the typodont phantom case, the proposed method has shown its out-performance to the intraoral optical method. Considering strong dependencies of the intraoral optical method on the surface reflectivity of the dental target area and also partly on the user experience, we envision that the advantages of the proposed method include not only dimensional accuracy but perhaps more importantly its robustness against dental surface states and users.

In addition, we would like to note that the proposed method is also very competent against the extraoral optical method as well. One does not have to suffer from laborious processes of the conventional method such as plaster pouring, sawing, and mounting. The dimensional discrepancy that can arise as a result of manual handling of stone model can also be mitigated, and overall turn-around time for dentists and patients can be reduced between preparation of the tooth and delivery of the restoration.

Although it has not been addressed in the results part, air bubbles can be trapped inside the dental impression and can leave unwanted scattered structures after segmenting air regions of the CT image. We utilized the Geomagic software to remove those air bubble structures in this work. A fully automatic image processing that converts the micro-CT image into the digital cast model would be desirable and is going to be developed as our future work. Our future work also includes clinical case studies to demonstrate its adequacy for clinical applications and to evaluate its clinical values in various aspects.

## Additional Information

**How to cite this article:** Kim, C. *et al*. Efficient digitalization method for dental restorations using micro-CT data. *Sci. Rep.* **7**, 44577; doi: 10.1038/srep44577 (2017).

**Publisher's note:** Springer Nature remains neutral with regard to jurisdictional claims in published maps and institutional affiliations.

## Figures and Tables

**Table 1:** Scanning parameters and system geometry in this work.

Parameter	Value
Voltage	60 kVp
Current	100 uA
Exposure time	150 mSec
Number of projection view	540
Detector matrix size	1944 × 1536
Detector pixel size	0.0748 mm
Image voxel size	0.0832 mm
Source-to-isocenter distance	211.3 mm
Source-to-detector distance	352.4 mm

**Table 2:** Measurements of the size length of each phantom.

		Degree: mm		
Type		Extraoral optical	Intraoral	Proposed
Phantom #1	True	7.00		
	Mean ± SD	6.987 ± 0.007	6.981 ± 0.009	6.984 ± 0.009
	95% CI	6.982–6.991	6.975–6.987	6.979–6.990
Phantom #2	True	8.00		
	Mean ± SD	7.987 ± 0.008	7.984 ± 0.008	7.984 ± 0.009
	95% CI	7.982–7.992	7.979–7.989	7.979–7.990
Phantom #3	True	4.00		
	Mean ± SD	3.988 ± 0.009	3.985 ± 0.007	3.986 ± 0.007
	95% CI	3.982–3.993	3.981–3.989	3.981–3.990

**Table 3:** Statistical results of the deviation map analysis.

		Type	
Metric (Degree: mm)		Intraoral	Proposed
Distance	Positive	0.1446	0.0617
	Negative	−0.0869	−0.0527
Standard deviation		0.1471	0.0839
